# Novel Quick Cell Patterning Using Light-Responsive Gas-Generating Polymer and Fluorescence Microscope

**DOI:** 10.3390/mi13020320

**Published:** 2022-02-18

**Authors:** Hidetaka Ueno, Yoshinori Akagi, Shohei Yamamura

**Affiliations:** 1 Health and Medical Research Institute, National Institute of Advanced Industrial Science and Technology (AIST), 2217-14 Hayashi-cho, Takamatsu 761-0395, Kagawa, Japan; ueno-hidetaka@aist.go.jp; 2 R&D Center Corporate Advanced Technology Institute Life Science Development Center, Sekisui Chemical Co., Ltd., 2-1 Hyakuyama, Shimamoto-cho 618-0021, Osaka, Japan; yoshinori.akagi@sekisui.com

**Keywords:** light-responsive gas-generating polymer, cell patterning, cell chips, functional polymers, cell microarray, lab-on-a-chip

## Abstract

Conventional cell patterning methods are mainly based on hydrophilic/hydrophobic differences or chemical coating for cell adhesion/non-adhesion with wavering strength as it varies with the substrate surface conditions, including the cell type and the extracellular matrix components (ECMs) coating; thus, the versatility and stability of cell patterning methods must be improved. In this study, we propose a new cell patterning method using a light-responsive gas-generating polymer (LGP) and a conventional fluorescence microscope. Herein, cells and cellular tissues are easily released from the substrate surface by the nitrogen gas bubbles generated from LGP by the excitation light for fluorescence observation without harming the cells. The LGP-implanted chip was fabricated by packing LGP into a polystyrene (PS) microarray chip with a concave pattern. HeLa cells were spread on the LGP-implanted chips coated with three different ECMs (fibronectin, collagen, and poly-D-lysine), and all HeLa cells on the three LGP patterns were released. The pattern error between the LGP pattern and the remaining HeLa cells was 8.81 ± 4.24 μm, less than single-cell size. In addition, the LGP-implanted chip method can be applied to millimeter-scale patterns, with less than 30 s required for cell patterning. Therefore, the proposed method is a simple and rapid cell patterning method with high cell patterning accuracy of less than the cell size error, high scalability, versatility, and stability unaffected by the cell type or the ECM coating.

## 1. Introduction

Cell patterning has been a key technology in cell biology and cell engineering for decades, especially drug discovery and evaluation, because it can control the adhesion area of cells, creating a cellular tissue model with any volume or shape [1]. Micro-scale cell patterning technology allows the observation of basic scientific biological behaviors, including cell polarity [2], cell division axis [3], or cell communication [4,5,6]. In addition, micro- to centimeter-scale patterning can be applied to drug screening. For example, the Organs-on-a-Chip technology to reproduce the metabolic functions of the human body on a palm-sized chip requires the construction of cellular tissues, mimicking the human organs, in equal proportions to the size of each human organ [7,8,9,10,11]. Unlike methods using gels to spatially confine cell tissues and restricting their growth and migration, cell patterning on a flat (2D) substrate does not physically control the cell culture space. Therefore, cell patterning on a substrate is also important in the study of cell or cell tissue metabolism, stem cells, and developmental biology, because it does not inhibit the expression of original cell functions by affecting the cell fate [12,13].

Cell patterning methods require accuracy of patterning, low load to cells, rapidity, versatility, and stability such that the patterning accuracy is not affected by the cell type, condition, and ECM coating, etc. Conventional cell patterning methods include physical patterning methods (direct manipulation wherein cells are directly placed, microdevices that sperate cells using 3D structures) and the surface modification method in which cells are cultured on the modified surface of a substrate. In the direct manipulation methods, cells are ejected from nozzles such as micro-capillaries and placed at designed positions [14]. In microdevices methods, cell patterning is performed by microstructure on various material substrates [15,16,17]. Although the cell patterning can be performed at the single-cell level using these microdevices, they load a physical stress on the captured cells due to the pressure, surface tension, and shear force. It is also difficult to release or cultivate the captured cells. Conversely, the surface modification methods that modify the surface on the culture substrate with chemical materials use hydrophilic/hydrophobic differences or chemical coating of cell-adhesive/non-adhesive materials. In the method using hydrophilic/hydrophobic differences, the surface of the substrate is modified via plasma treatment, etc., to change the adhesion force with cells and create cell adhesion and non-adhesion areas [18]. Chemical coating methods are performed by transferring the cell adhesion materials to micro-patterns using micro-sized structures as stamps, which is called microcontact printing [19,20], or by introducing materials for surface modification into the gap between the pressed micro-sized structures and the substrate [21]. Alternatively, the cell adhesion/non-adhesion substance material, once uniformly coated on the substrate, is partially removed and modified using UV [22,23,24]. Although various surface modification methods for cell patterning have been proposed as described above, it is still difficult to control cell adhesion versatility and stability only by hydrophilic/hydrophobic differences or chemical coating on the surface because the adhesion strength of cells differs among cell types and even individual cells. In addition, the morphology, proliferation rate, differentiation, and gene expression of cells are affected by the scaffold material to which they are attached. Therefore, to construct an appropriate culture environment for each kind of cell, the surface of the substrate on which cells are cultured must be coated with an ECM [25,26,27,28,29,30]. For example, when culturing adhesive cells, an ECM such as fibronectin or collagen must be used, but simultaneously using the ECM and chemical coated substances for cell patterning is extremely difficult because they overlap on the substrate and cancel each other’s effect. Previous research tried to accurate cell patterning with a coating multilayer that consists of two kinds of chemical materials for cell adsorbing and releasing [31,32]. However, it is extremely difficult to simultaneously use tissue-derived biomaterial (e.g., collagen, fibronectin, type of ECM) for cell adhesion and culture. Therefore, the surface modification method has issues of versatility and stability.

**Figure 1:**
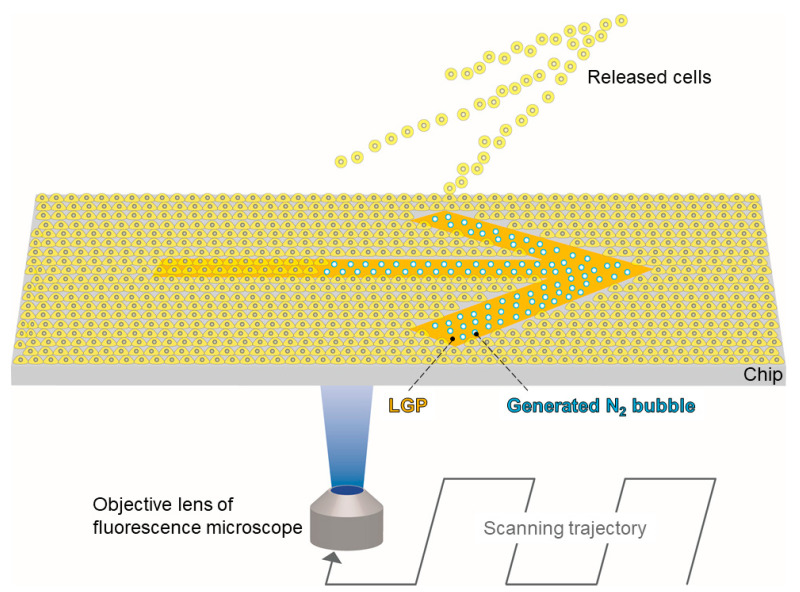
The principle of the cell patterning method using an LGP-implanted chip. After spreading cells on a polystyrene microarray chip implanted with LGP, it is irradiated with 365 nm excitation light from an inverted microscope to generate nitrogen gas. The physical force of the generated nitrogen gas causes the cells attached to the LGP surface to be released, and the remaining cell layer on the substrate is patterned. The proposed method can be applied to a pattern that is larger than the beam size of the objective lens by using the scanning function of an inverted microscope.

In our previous research, we developed an LGP coating technique for cell release using N_2_ bubble generation [33,34]. In the present study, we propose a novel, easy, and versatile cell patterning method using an LGP-implanted chip consisting of LGP and a microarray chip that generates nitrogen gas by light of a specific wavelength via a fluorescence microscope. In the LGP-implanted chip method, the nitrogen gas bubbles physically release the adherent cells. Because the cells are physically released by N_2_ bubbles, the substrate surface with the cells spread can be freely coated with various ECM molecules, such as fibronectin and collagen. By coating these molecules, the cell adhesion surface can be easily modified to obtain the most suitable conditions for adhesion and culture of various cells. In addition, the variation of the adhesion strength of the adherent cells does not affect the cell release. The principle of the proposed method is shown in Figure 1. LGP-implanted chips are fabricated by packing LGP into the injection-molded concave microarray chips made of polystyrene (PS). The LGP-implanted chip is coated with ECMs (fibronectin, collagen, and poly-D-lysine). After HeLa cells have adhered to the LGP patterns, nitrogen gas bubbles are generated from the LGP using excitation light (365-nm wavelength, UVA), commonly used for observations, such as nuclear staining by fluorescent microscope. The generated bubbles only release the HeLa cells on the LGP pattern on the substrate. After patterning, we evaluated the accuracy of the cell patterns.

## 2. Materials and Methods

### 2.1. Cell Culture

HeLa-H2B-green fluorescent protein (GFP) cells were employed as the cultured cell line. HeLa cells were human cervical cancer cells expressing GFP. The culture medium was prepared by mixing Dulbecco’s Modified Eagle’s Medium (DMEM, 12800-017, Thermo Fisher Scientific Inc., Waltham, MA, USA) with 10 v/v% fetal bovine serum (FBS, Merck KGaA, Darmstadt, Germany) and 5 v/v% penicillin (P4333, Merck KGaA, Darmstadt, Germany). The HeLa cells were cultured in a 5% CO_2_ atmosphere at 37 °C. Phosphate-buffered saline (PBS, T900, Takara Bio Inc., Shiga, Japan) was used as a cell washing solution. A solution of 0.11% Trypsin/1 mM EDTA (25200056, Thermo Fisher Scientific Inc., Waltham, MA, USA) was used to remove the cells adhered to the bottom of cell culture dish with the diameter of 90 mm (S90-NC18, Fine Plus International Ltd., Kyoto, Japan). For the experiment using the arrow pattern, HeLa cells were stained by CellBrite green (30021, Biotium Inc., CA, USA).

### 2.2. Fabrication Method for LGP-Implanted Chip

**Figure 2:**
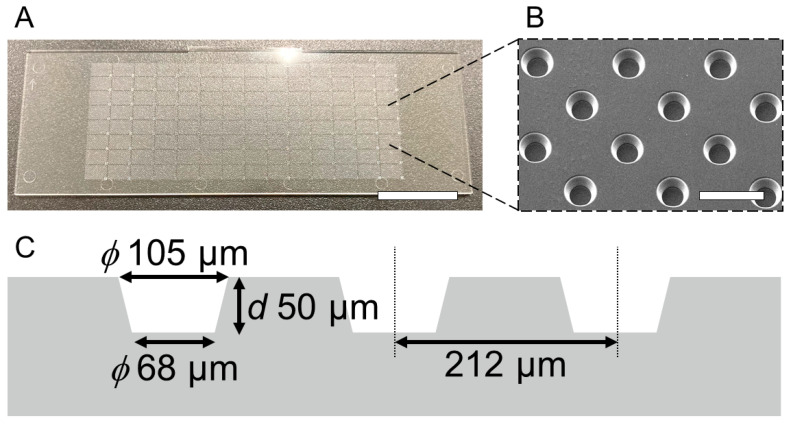
Design of the microarray chip made of polystyrene (PS). (**A**) Photograph of the microarray chip with 20,944 microchambers. The scale bar indicates 10 mm. (**B**) SEM image of PS microarray chip. Microchambers are arrayed at regular intervals. The scale bar indicates 200 μm. (**C**) Schematic image of the cross section of microarray chip. The top and bottom diameters of the microchamber are 105 μm and 68 μm, respectively. The depth of the chamber is 50 μm. The interval between the microchambers is 212 μm.

The LGP-implanted chip was fabricated by packing LGP into a PS microarray chip with multiple chambers designed in an array (SEIKOH GIKEN Co., Ltd., Chiba, Japan) [35,36,37]. The photographs and design values of PS microarray chips are shown in Figure 2. Each microarray chip was 76 mm wide, 25.5 mm long, and 1 mm thick (Figure 2A), with 20,944 equally spaced microchambers (Figure 2B). The microchambers were inverted cone trapezoids with an upper diameter of 105 μm, a lower diameter of 68 μm, and a depth of 50 μm. The microarray chip was fabricated by injection molding (Figure 2C).

**Figure 3:**
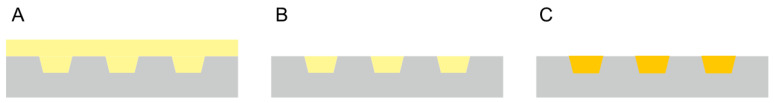
Fabrication process of the LGP-implanted chip. (**A**) LGP mixed with the main agent and crosslinker were deposited on the polystyrene microarray chip. (**B**) LGP on the microarray chip surface was removed. (**C**) Polymerization reaction of the LGP was performed at room temperature of 22 °C for over 24 h.

LGP is an azide compound polymer, losing its fluidity and becoming solid when mixed with a cross-linking agent. To form solid LGP, a main agent and a crosslinker were mixed at a 20:1 volume ratio. The main agent comprised glycidyl azide polymer (GAP; NOF Corporation, Tokyo, Japan) and photosensitizer (2-isopropylthioxanthone; Tokyo Chemical Industry Co., Ltd., Tokyo, Japan) mixed at a 100:3 volume ratio. The crosslinker was used as a curing agent (poly-2-(2-ethoxyethoxy)ethyl acrylate; Nippon Shokubai, Osaka, Japan). The fabrication process of the LGP-implanted chip is shown in Figure 3. The LGP was applied onto the microarray chip immediately after mixing the main agent and crosslinker (Figure 3A). Excess LGP was removed by scraping the microarray chip surface using a piece of silicone rubber sheet (Tigers Polymer Corporation, Osaka, Japan), and the microchamber was filled with LGP (Figure 3B). LGP was cured overnight at 22 °C (Figure 3C). The LGP became solid and stored for a long time under a light-shielded condition.

The fabrication accuracy, surface condition, and autofluorescence of the fabricated LGP-implanted chips were evaluated. The fabrication accuracy and surface condition were observed and measured by optical microscope (VHX-5000; Keyence Co., Ltd., Osaka, Japan), scanning electron microscope (SEM, JSM-6060-EDS, JEOL Ltd., Tokyo, Japan), and white light interferometer (NT91001A-in motion; Bruker Japan, Tokyo, Japan). Surface roughness was evaluated by the calculated arithmetical mean height (Ra) and the maximum profile height (Rt) [38]. The autofluorescence of HeLa cells, LGP, and PS surfaces were measured using ImageJ software (NIH).

### 2.3. Cell Releasing from the LGP-Implanted Chip Coated with Fibronectin, Collagen, and Poly-D-Lysine

**Figure 4:**
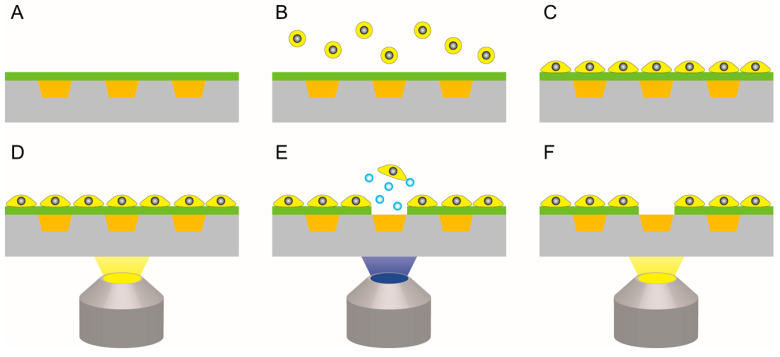
Cell releasing process from the LGP-implanted chip. (**A**) The LGP-implanted chip was coated with each coating material (fibronectin, collagen, poly-D-lysine). (**B**) HeLa cells were spread on the chip. (**C**) HeLa cells were attached to the chip. (**D**) Adhered HeLa cells were observed by fluorescence microscopy using 488 nm excitation light. (**E**) The filter was switched, and nitrogen gas bubbles were generated from the LGP using 365 nm excitation light to release the HeLa cells from the LGP. (**F**) The filter was switched back to the 488 nm excitation light and the remaining HeLa cells were observed.

HeLa cells were released from the surface of the LGP-implanted chip coated with fibronectin, and cell patterning accuracy was evaluated by comparing the difference of the LGP and the cell layer patterns. The cell release process is shown in Figure 4. First, the LGP-implanted chip was coated with fibronectin (F1141, Merck KGaA, Darmstadt, Germany). The concentration of fibronectin was adjusted to 20 μg/mL using pure water. After the fibronectin solution was dropped on the LGP-implanted chip, it was left in a refrigerator at 4 °C for 24 h. After washing the LGP-implanted chip with PBS, one ml of HeLa cell suspension (1 × 10^7^ cells/mL) was dropped onto the chip and incubated in a CO_2_ incubator (37 °C, CO_2_: 5%) for 1 h. After incubation, the chip was rinsed in the medium to remove any unattached cells. The LGP-implanted chip was placed on the stage of an inverted fluorescence microscope (IX-73, Olympus, Tokyo, Japan) with a CCD camera (DP80, Olympus, Tokyo, Japan) to confirm that the HeLa cells attached and covered the surface of the chip by using the filter (U-FBNA; Olympus, Tokyo, Japan). We used the objective lens (magnification; 10, LMPLFLN10X, Olympus, Tokyo, Japan) with a beam size diameter of 2.4 μm at 488 nm and 1.8 μm at 365 nm. We focused on a specific microchamber filled with LGP covered by HeLa cells and switched the filter from the irradiating 488 nm excitation light (U-FBNA; Olympus, Tokyo, Japan) to that irradiating 365-nm excitation light (U-FUNA; Olympus, Tokyo, Japan). Nitrogen gas bubbles were generated from LGP irradiated with the 365-nm light, releasing the cells on the LGP. The filter was switched back to the filter (U-FBNA; Olympus, Tokyo, Japan) irradiating 488 nm excitation light, and the surface of the LGP-implanted chip was observed after the HeLa cells were released. Similar experiments were also performed using collagen from calf skin (C9791, Merck KGaA, Darmstadt, Germany) with the concentration adjusted to 100 μg/mL and poly-D-lysine (P7280, Merck KGaA, Darmstadt, Germany) with the concentration adjusted to 50 μg/mL.

The area of the cell layer released from the fibronectin-coated LGP-implanted chip was measured using ImageJ, and its area was determined assuming that it was circular. The difference between the microchamber radius and the radius of the area where the cells were released was calculated as the patterning error from Equation (1).

(1) $$\mathrm{Patterning}\;\mathrm{error}=\sqrt{\frac{S}{\pi }}-r\;(\mu m)$$

where *S* indicates the area of released cells and was measured by ImageJ, *π* is the circumference, and *r* is the opening radius of the microchamber. The average and standard deviation were calculated for nine LGP-implanted chambers.

To evaluate the patterning accuracy and required time of the LGP-implanted chip method for large patterns, we performed cell patterning using a 1.5 × 0.9 mm arrow pattern consisting of 100 μm wide lines and 60 μm depth. The LGP was implanted in a concaved arrow pattern via the same process as that for implanting it into the microchamber on the microarray chip. A suspension of HeLa cells was dropped onto the arrow pattern coated with fibronectin and incubated in a CO_2_ incubator (37 °C, CO_2_: 5%) for 1 h. After incubation, the LGP-implanted chip was rinsed in the medium to remove any unattached cells. The LGP-implanted chip was placed on the stage of an inverted fluorescence microscope (IX-73, Olympus, Tokyo, Japan) with a CCD camera (DP80, Olympus, Tokyo, Japan). We used the objective lens (magnification; 10, LMPLFLN10X, Olympus, Tokyo, Japan) with beam size diameter of 2.4 μm at 488 nm and 1.8 μm at 365 nm. It was confirmed that the HeLa cells covered the entire surface of LGP-implanted arrow pattern by using auto-scanning of fluorescent microscope with the filter (U-FBNA; Olympus, Tokyo, Japan) irradiating 488 nm light. Next, we switched to the filter (U-FUNA, Olympus, Tokyo, Japan) irradiating 365-nm light and auto-scanned the entire arrow pattern again. Since the LGP was irradiated with the excitation light at 365 nm, nitrogen gas bubbles were generated and HeLa cells on the LGP were released. After switching to the filter (U-FBNA; Olympus, Tokyo, Japan) irradiating 488 nm light, the arrow pattern and the cell layer were observed by the auto-scanning function of the fluorescence microscope.

## 3. Results and Discussion

### 3.1. Characterization of LGP-Implanted Chip

**Figure 5:**
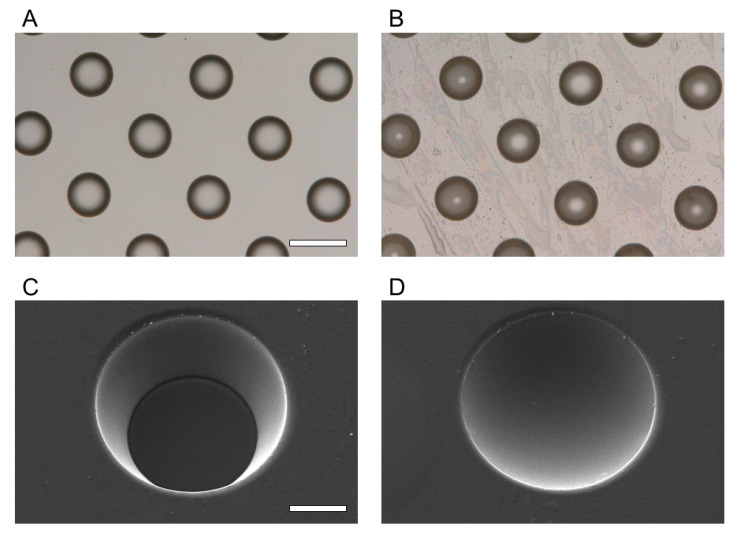
Images of the PS microarray chip before and after LGP packing. (**A**,**B**) Microscopic image of the surface of the microarray chip before and after LGP implantation, taken with an optical microscope. (**C**,**D**) SEM image of the chamber on the microarray chip before and after LGP implantation. The scale bar in (**A**,**B**) is 100 μm, and the scale bar in (**C**,**D**) is 30 μm.

**Figure 6:**
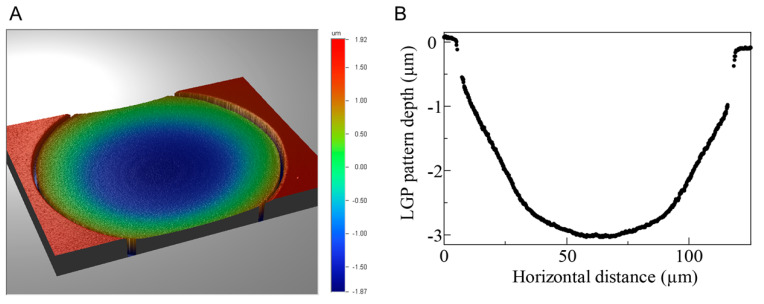
LGP-implanted microchamber measured by the white light interferometer. (**A**) Three-dimensional profile image of the microchamber surface. (**B**) The height difference of the LGP in the microchamber.

The fabricated LGP-implanted chips were evaluated for shape and surface condition using an optical microscope (VHX-5000, Keyence Co., Ltd., Osaka, Japan), SEM (JSM-6060-EDS, JEOL Ltd., Tokyo, Japan), and white light interferometer (NT91001A-in motion; Bruker Japan, Tokyo, Japan). The microscopic images of the microchambers before and after filling with LGP are shown in Figure 5A,B, respectively. After the LGP was implanted, light reflection from inside the microchamber was observed. Figure 5C,D show the SEM images of the microchambers before and after filling with LGP, respectively. It was observed that the microchambers were filled with LGP after the implantation process. Figure 6 shows the result of observation by the white light interferometer. The edge and areas around of the microchamber was not observed as white light interference was not obtained. The maximum height difference between the LGP surface and the PS surface was approximately 3 μm. The arithmetical mean height (Ra) was 0.54 ± 0.04 μm, and the maximum profile height (Rt) was 2.24 ± 0.23 μm. As the size of the HeLa cells was approximately 10–15 μm, the fabrication error (Rt: 2.24 ± 0.23 μm) of the LGP-implanted chip was within the range not affecting the cell observation.

**Figure 7:**
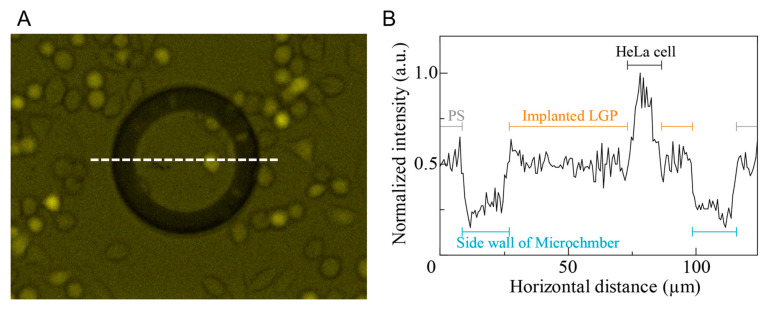
Comparison of fluorescence images of HeLa cells, implanted LGP and PS substrate. (**A**) Fluorescence images of a HeLa cell spread on the LGP-implanted chip. (**B**) Normalized fluorescence intensity on the line in the fluorescence image.

HeLa cells were spread on the LGP-implanted chip and observed by fluorescence microscopy. The fluorescent image is shown in Figure 7A. In the image, GFP-expressed HeLa cells were observed on both the LGP-implanted microchambers and PS surface. The normalized line intensity in the image was measured by ImageJ (Figure 7B) with the maximum intensity obtained in HeLa cells. As the LGP fluorescence intensity was comparable to that of PS substrate used for general cell culture, and approximately half the intensity of HeLa cells, it was indicated that the autofluorescence of the LGP-implanted in the PS chip had no negative effect on the cell observation.

The LGP-implanted chip was fabricated by packing LGP into a 76 × 25.5 mm PS concave microarray chip fabricated via injection molding. The LGP implantation into the PS chip was performed manually without any special equipment. The cross-linking reaction occurred at room temperature (22 °C), thus there was no need for strict temperature and humidity control using special equipment. The fabrication accuracy of the LGP-implanted chip was too small to interfere with the cell observation. Therefore, the process of LGP implantation was simple with high yield because it did not require expensive equipment or strict manipulation. In addition, after cross-linking, as LGP is an extremely stable material with a heat resistance temperature of approximately 100 °C, it can be stored for a long time under room temperature. Therefore, LGP-implanted chips, fabricated by packing LGP into PS microarray chips can be modified with various LGP patterns and are superior in terms of mass production and long-term storage.

### 3.2. Cell Patterning by LGP-Implanted Chip Coated by Fibronectin, Collagen, and Poly-D-Lysine

**Figure 8:**
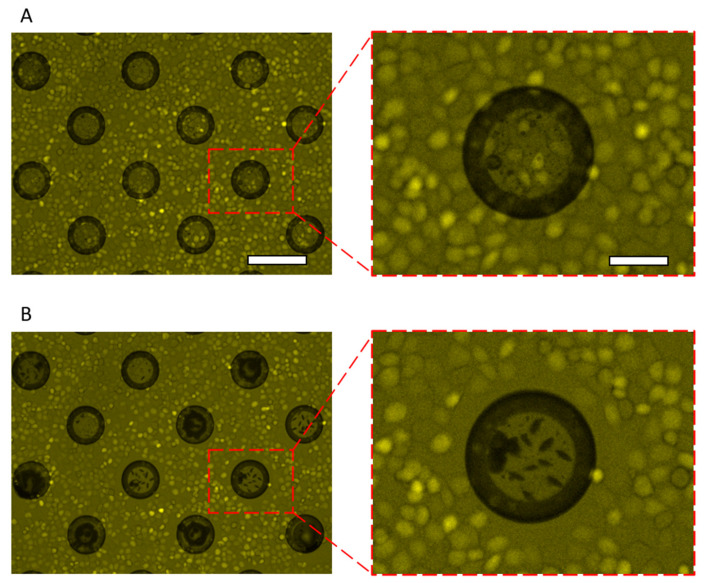
Fluorescence images of the HeLa cells released from a fibronectin-coated LGP-implanted chip. (**A**) Image of HeLa cells on the surface of LGP-implanted chip coated with fibronectin. (**B**) Images of HeLa cells on the surface of the LGP-implanted chip after 365 nm light irradiation. All HeLa cells on the LGP and some HeLa cells around the LGP were released by nitrogen gas bubbles. Right side pictures show magnified images in the boxes. The scale bars indicate 150 μm in the wide images and 50 μm in the magnified images.

**Figure 9:**
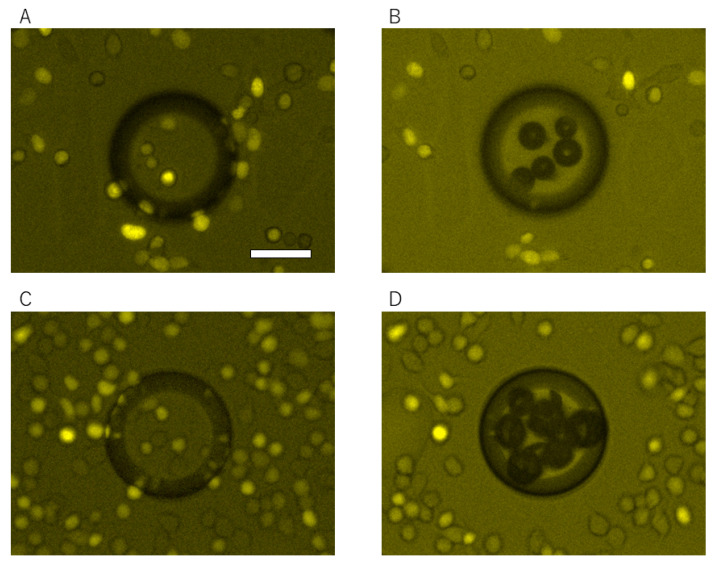
Fluorescence images of the LGP-implanted chip surface before and after HeLa cells released by N_2_ gas generation with the 365-nm excitation light. (**A**,**B**) Collagen-coated LGP-implanted chip surface before and after excitation light irradiation. (**C**,**D**) Poly-D-lysine-coated LGP-implanted chip surface before and after excitation light irradiation. HeLa cells were released from the LGP surfaces regardless of the coating material. The scale bar is 50 μm.

HeLa cells were spread on the LGP-implanted chip coated with fibronectin and incubated for 1 h, then the cells were released by generating nitrogen gas exposed with 365-nm light. HeLa cells adhered to the entire surface of the LGP-implanted chip without any gaps (Figure 8A), and only those on the LGP-implanted microchambers were completely released after irradiation with 365-nm excitation light (Figure 8B). HeLa cells on the LGP surface were released completely and partially from the surrounding area by spreading nitrogen gas bubbles there. The same operation was performed for nine chambers, and the excess release range was calculated using Equation (1). The calculated excess of the release range was 8.81 ± 4.24 μm outward from the boundary of the chamber wherein the LGP was implanted. As the size of a HeLa cell is generally approximately 10–15 μm, the pattern error in cell releasing using LGP was smaller than one cell size. Therefore, it was shown that the accuracy of the proposed method was of the single-cell size level. Similar experiments were also performed using collagen from calf skin (C9791, Merck KGaA, Darmstadt, Germany) and poly-D-lysine (P7280, Merck KGaA, Darmstadt, Germany), and the results are shown in Figure 9. At least five or more cells were attached on LGP-implanted microchambers before the 365-nm excitation light (Figure 9A,C). After the excitation light was irradiated and nitrogen gas bubbles were generated from the LGP, HeLa cells were completely released from the LGP-implanted area, regardless of the coating materials (Figure 9B,D). The time required for releasing HeLa cells was less than 10 s, including the filter switching time. Furthermore, there was no damage to the adhered, released, or remaining HeLa cells on the substrate.

Because the amount of nitrogen gas generated and the physical force to release those cells were not affected by the cell type or the coating material, the LGP-implanted chip method may work with different kinds of cells and coating materials. In addition, the cells were released by simply switching the filter, which was extremely rapid and easy compared to other methods. Furthermore, it is possible to observe the condition of the cells before and after patterning by switching the filter and confirm in real time the formation of the designed pattern.

**Figure 10:**
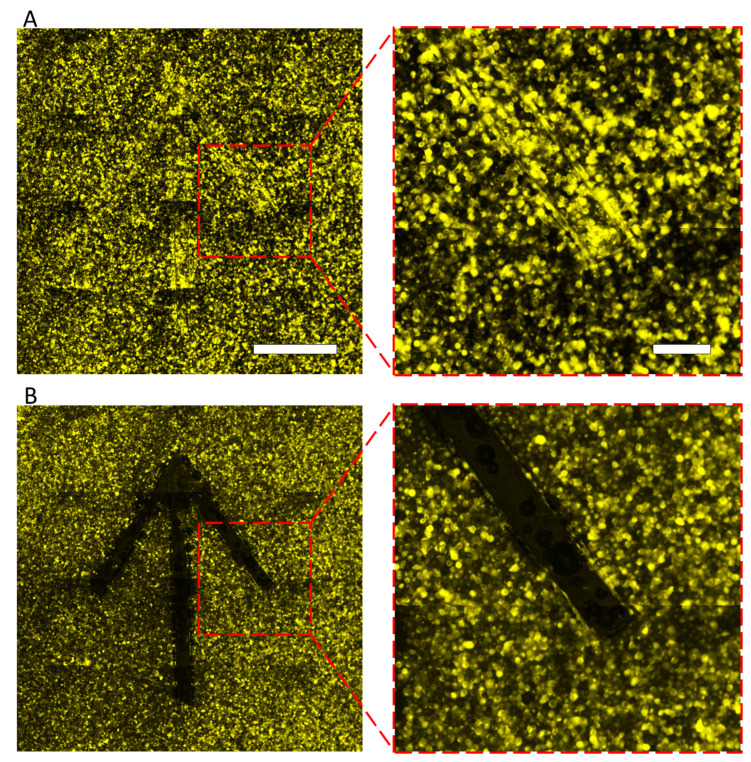
Fluorescent images of the HeLa cell patterning on the arrow shape. (**A**) Arrow pattern implanted with LGP and its surrounding area after spreading HeLa cells. (**B**) Fluorescence image of the arrow pattern after nitrogen gas generation by 365 nm light irradiation. Only HeLa cells attached to the LGP were released, and the remaining cells showed the shape of the same to the original arrow pattern. The scale bar indicates 500 μm in the wide area image and 100 μm in the magnified image.

To confirm the patterning accuracy at the millimeter-scale, an arrow pattern was fabricated via the proposed method. Figure 10 shows the fluorescence images of HeLa cells spread and released on the LGP-implanted chip with the arrow pattern. As the cells were spread on the chip, they adhered to both the LGP and PS surfaces without any gaps (Figure 10A). After irradiating with the 365 nm light, the HeLa cell fluorescence was not observed on the LGP, and the remaining cells on the chip had the same arrow shape as the LGP pattern (Figure 10B). The entire pattern was irradiated with 365-nm excitation light for less than 30 s (each spot was irradiated for 30 ms). Therefore, the proposed method enabled simple, rapid, and accurate cell release and patterning even at the millimeter scale.

**Figure 11:**
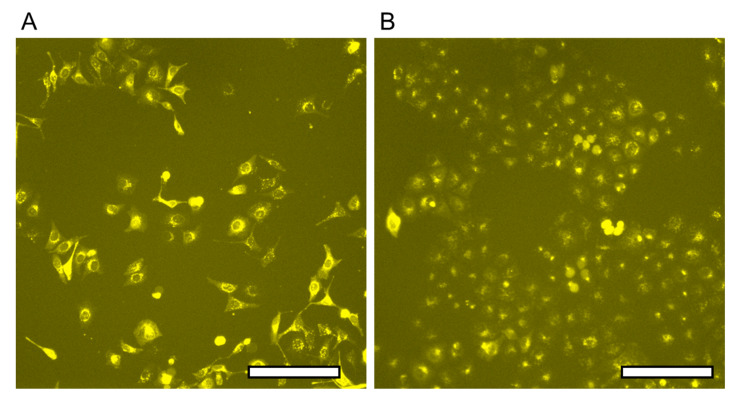
Fluorescent image of HeLa cells released from the LGP-implanted chip. (**A**) Hela cells after a day of cultivation. (**B**) Hela cells after three days of cultivation. The scale bar indicates 20 μm.

The fluorescent image of released HeLa cell cultivation is shown in Figure 11. The released cells adhered and extended in the 96 well plates within one day, and then they made enough growth for 3 days. This indicated that the proposed method does not cause damage to cells by nitrogen gas and light irradiation. The 365 nm light is long-wave UV light (UVA, 315–400 nm), which is totally different from 250 nm light used for normal UV sterilization lamps, and appears to be generally non-toxic. Additionally, the total exposure energy was 3.68 mJ/cm^2^ in this experiment. It was expected that there was no damage to cells by the irradiation light because reported research showed no damage to cell cultivation under 5.4 mJ/cm^2^ exposure [39]. Therefore, the proposed method is not harmful to either the cells remaining on the LGP-implanted chip or the cells released from the chip. 

In this experiment, nitrogen gas bubbles were generated using a standard inverted fluorescence microscope (IX-73; Olympus, Tokyo, Japan) and a filter (U-FUNA; Olympus, Tokyo, Japan, 365-nm) used for fluorescence observation using standard fluorescent reagents, such as nuclei stains: DAPI and Hoechst. Moreover, no additional equipment was required to use the proposed method. In addition, as bubbles are generated by the excitation light of a specific wavelength irradiated from the microscope, cell patterning and observation can be done in parallel simply by switching the filter. In the patterning of the millimeter-scale arrow pattern, the excitation light for gas generation was irradiated for 30 s using the auto-scanning function of the microscope. Although the scanning range differs among microscope models, the LGP-implanted chip method can be extended not only to the millimeter-scale but also to the centimeter-scale because it generally scans several centimeters. Therefore, the LGP-implanted chip method does not require special equipment, does not cause cell damage, and is simple, fast, and accurate with sufficient scalability. In this paper, we have shown patterning by releasing cells, but it could also be applied to screening defective cells and retrieving specific cells. Therefore, it is expected to be used not only for patterning as a cell culture and evaluation, but also for high-throughput screening of cells.

## 4. Conclusions

In this study, we proposed a novel easy, rapid, and versatile cell patterning method using the LGP and excitation light of a general fluorescence microscope. As the LGP-implanted chip method can physically release cells, we completely released HeLa cells from the LGP surface coated with either fibronectin, collagen, or poly-D-lysine. The pattering error was 8.81 ± 4.24 μm, smaller than single-cell size. By using the auto-scanning function of the inverted microscope, millimeter-scale cell patterns were formed within 30 s. This method can be applied directly for the large (centimeter)-scale cell patterns without any additional equipment. Moreover, the cell release by the proposed method is less damaging to the cells, thus it can be applied to retrieve and screen specific cells in the future. Furthermore, as the LGP-implanted chip method solely requires a standard fluorescence microscope, it is simple, rapid, and has higher versatility and stability as compared to other cell patterning methods.
